# Factors Used by Mobile Applications to Predict Female Fertility Status and Their Reported Effectiveness: A Scoping Review

**DOI:** 10.7759/cureus.48847

**Published:** 2023-11-15

**Authors:** Elaine E Saugar, Sabine Katsoulos, Hyun-su Kim, Nazanin Fakharzadeh, Jacob Schaffer, Maubeen Ahmad, Caitlin Zeher, Meghan Benedict, Sarina Gupta, Gina Foster-Moumoutjis

**Affiliations:** 1 Dr. Kiran C. Patel College of Osteopathic Medicine, Nova Southeastern University, Fort Lauderdale, USA; 2 Dr. Kiran C. Patel College of Osteopathic Medicine, Nova Southeastern University, Clearwater, USA; 3 Department of Family Medicine, Nova Southeastern University Dr. Kiran C. Patel College of Osteopathic Medicine, Davie, USA

**Keywords:** female sexual and reproductive health, digital health self-tracking, ovulation, family planning, conception, female contraception, fertility awareness-based method, fertility tracking app, fertility, mobile application

## Abstract

Family planning, whether for pregnancy prevention or conception, is of pivotal importance to women of reproductive age. As hormonally driven methods, such as oral contraceptive pills, are widely used but have numerous side effects, women often seek alternative non-hormonal, non-invasive options, including fertility-tracking mobile applications (apps). However, the effectiveness of these apps as a method of contraception and conception planning has not been extensively vetted. The goal of this scoping review is to identify the various factors used by apps marketed as a method of contraception and/or family planning to predict a woman’s fertility status, as well as their documented effectiveness.

Following the Preferred Reporting Items for Systematic Reviews and Meta-Analyses Extension for Scoping Reviews guidelines, a literature search was performed in CINAHL, MEDLINE, and Alt HealthWatch databases for articles published between October 1, 2017, and October 4, 2022. Quality assessment of eligible full-text articles was conducted using the Joanna Briggs Institute critical appraisal tools.

A total of 629 articles were screened. Overall, 596 articles were excluded and the remaining 33 articles underwent full-text review. Seven articles were included in the final analysis, yielding data on the following five apps: Natural Cycles, Ava Fertility, Clearblue Connected, Ovia Fertility, and Dynamic Optimal Timing (DOT).

Data supporting the effectiveness of these apps is limited. All apps provided predictions on fertility status throughout a woman’s menstrual cycle using proprietary algorithms, biometric data, and self-reported menstrual cycle data. Further research, particularly independent research following a randomized controlled design, on the efficacy of these apps is needed to produce more robust results.

## Introduction and background

Unintended pregnancy poses risks for both the mother and child. Women who experience an unintended pregnancy are more likely than those with a planned pregnancy to receive inadequate or delayed prenatal care, use tobacco or alcohol during pregnancy, and are less likely to breastfeed; these factors lead to a higher risk of prematurity, low birth weight, and long-term physical and mental health problems [1]. According to the United Nations Department of Economic and Social Affairs, it was estimated in 2019 that among the 1.9 billion women of reproductive age (15-49 years) worldwide, 1.1 billion had a need for family planning and 190 million did not have this need met [2]. Additionally, the disparities in access to resources for family planning are largest in low- and lower-middle-income countries [2]. Moreover, in some countries, like Iran, permanent contraceptive methods, such as vasectomies and tubectomies, have been outlawed and access to contraception is strictly restricted by the government [3]. These limitations lead to an increase in the number of unintended pregnancies and unsafe abortions globally. According to the World Health Organization (WHO), unsafe abortion is a leading cause of maternal morbidity and mortality, accounting for 45% of all abortions, of which 97% occur in developing countries [4]. Interventions that increase access to effective, low-cost methods of contraception and family planning are needed to address this global health issue of unintended pregnancy.

According to the United States (U.S.) Centers for Disease Control and Prevention, oral contraceptive pills (OCPs) are the most used form of non-barrier, reversible birth control in the U.S. in women ages 15 to 49 [5]. Although OCPs are a popular form of birth control, they have numerous side effects, such as headaches, mood swings, and irritability. A study found that 75% of Jordanian women using OCPs reported experiencing these side effects, and a majority of the women who elected to discontinue taking them cited the unwanted side effects as the primary reason for doing so [6]. Alternatives to hormonal contraception include condoms, sponges, spermicidal agents, diaphragms, and the withdrawal method. While the latter is often attempted due to its cost-effectiveness and ease of use, withdrawal as a method of contraception has an almost 20% failure rate [7,8]. Cycle tracking using a mobile application (app) may provide the same convenience and lower contraceptive failure rate with recommended use [9].

Smartphone usage has become ubiquitous, even crossing socioeconomic barriers [10], and provides access to a multitude of apps through various platforms such as Apple iTunes and Google Play. There are over 165,000 health apps in the U.S. Apple iTunes and Google Play stores alone [11]. Given the recent shift in the way women prefer to track their menstrual cycle, from using traditional calendar-based methods to more sophisticated fertility-tracking apps [12], app companies have developed proprietary algorithms to predict fertile window and ovulation; however, these algorithms are often not supported by evidence-based research, and thus their efficacy is unknown [13].

Knowledge of the female reproductive cycle has increased and there is now a greater understanding of the symptomatology present periodically throughout the menstrual cycle and various physiological markers of ovulation. As there are several different metrics to estimate ovulation dates, the required user input and method of calculation vary from app to app; some apps use biometric data, such as basal body temperature (BBT) [14] and urine luteinizing hormone (LH) level, while others solely rely on self-reported cycle history and subjective symptoms (e.g., characteristics of cervical secretions) to provide predictions [15]. Identifying which apps provide the most reliable predictions and what factors they use to make these predictions would be valuable information for healthcare providers as they counsel and educate their patients. In addition, fertility-tracking apps have the potential to help women avoid unintended pregnancy and have more precision when family planning.

The purpose of this scoping review is to explore the various factors used by apps marketed as a method of contraception and/or family planning currently available worldwide to predict female fertility status, as well as any reported effectiveness data. The scoping review published in 2020 by Earle et al. provided a broad overview of the use of menstruation and fertility-tracking apps, focusing on women’s motivations for using them. They reported that, regardless of the motive for using an app, users’ primary concern was the predictive accuracy of the app [13]. While there are several articles detailing specific apps and their effectiveness [14,15], to our knowledge, this information has not been compiled into a review that compares the reported effectiveness of several fertility-tracking apps, as well as the type of user data each requires to provide predictions. This review intends to provide healthcare providers with a reference that will aid them in making recommendations to their patients interested in using this technology for contraception or family planning.

This article was previously presented as a poster and abstract at the Nova Southeastern University Dr. Kiran C. Patel College of Osteopathic Medicine Office of Graduate Medical Education (NSU-KPCOM OGME) Student/Intern/Resident/Fellow Scientific Research Poster Competition on April 21, 2023.

## Review

Search strategy

This scoping review followed the Preferred Reporting Items for Systematic Reviews and Meta-Analyses (PRISMA) Extension for Scoping Reviews guidelines [16]. To streamline the research process and avoid variability in the search strategy for each database, EBSCOhost was used. Within EBSCOhost, three databases, namely, CINAHL, MEDLINE, and Alt HealthWatch, were searched for articles published in academic journals between October 1, 2017, and October 4, 2022, in English, and in the U.S. The databases were chosen based on the number of journals indexed, as well as their relevance to the research question. CINAHL includes literature from the fields of nursing and allied health. MEDLINE registers an extensive variety of publications within medicine and clinical sciences. Alt HealthWatch features research on complementary and alternative medicine. The list of search terms was created based on a search done on the U.S. National Library of Medicine’s Medical Subject Headings browser and a review of the search strategy by Earl et al. [13]. Using Boolean operators, search terms were combined as follows: ovulation OR “menstrual cycle” OR “fertility awareness” OR contraception AND (“mobile application” OR “mobile app” OR “smartphone app” OR “smartphone application” OR “cell phone application” OR “cell phone app” OR mhealth OR “m-health” OR “mobile health”) AND (women OR female OR woman) AND (“self-tracking” or “self tracking”) AND predict NOT (surgery or surgical) NOT (“polycystic ovary syndrome” OR PCOS OR “polycystic ovarian syndrome” OR “polycystic ovaries”) NOT (“in vitro fertilization” or IVF).

Eligibility criteria

To meet the inclusion criteria, articles needed to have discussed at least one smartphone-accessible app marketed as a method of contraception and/or aid in family planning. Studies that discussed an app not available on smartphones or apps solely used for education purposes (i.e., it only provides information on methods of contraception and family planning) were excluded given the focus of the research question. In addition, compatibility with a specific smartphone operating system, region-locked status, language of the interface, or endorsement by a medical institution were not factors that limited inclusion to prevent excessive restriction of the search results. While data on the efficacy of mobile applications was collected, the absence of this data in a publication did not exclude it from this review, given the primary endpoint was to determine the factors used by mobile applications. Regarding the study population, only studies on women of reproductive age (15 to 49 years old) were included; however, those solely comprising female athletes were excluded due to poor generalizability and the possibility of skewing the analysis. Other demographic characteristics, such as race, ethnicity, and socioeconomic status, as well as the geographic location of the study population, did not limit inclusion. Additionally, only original studies, such as randomized controlled trials, exploratory, cohort, prospective, feasibility, or qualitative assessments, were included. Lastly, case reports, reviews, editorials, viewpoints, guidelines, letters to editors, commentaries, and publications that were not peer-reviewed were excluded.

Data items and charting

Data abstracted from the seven studies included in this scoping review were organized into a table on a Microsoft Word document and consisted of the following: author(s), date of publication, study design and aims, description of the study population, specific app studied and any required accessory device, information used by the app to predict fertility, reported effectiveness, marketed purpose of the app (i.e., contraception, conception, or both), potential conflicts of interest, and study limitations. Any doubts regarding the eligibility of the studies brought up during the charting process were resolved through group discussion.

Screening and critical appraisal

**Figure 1:**
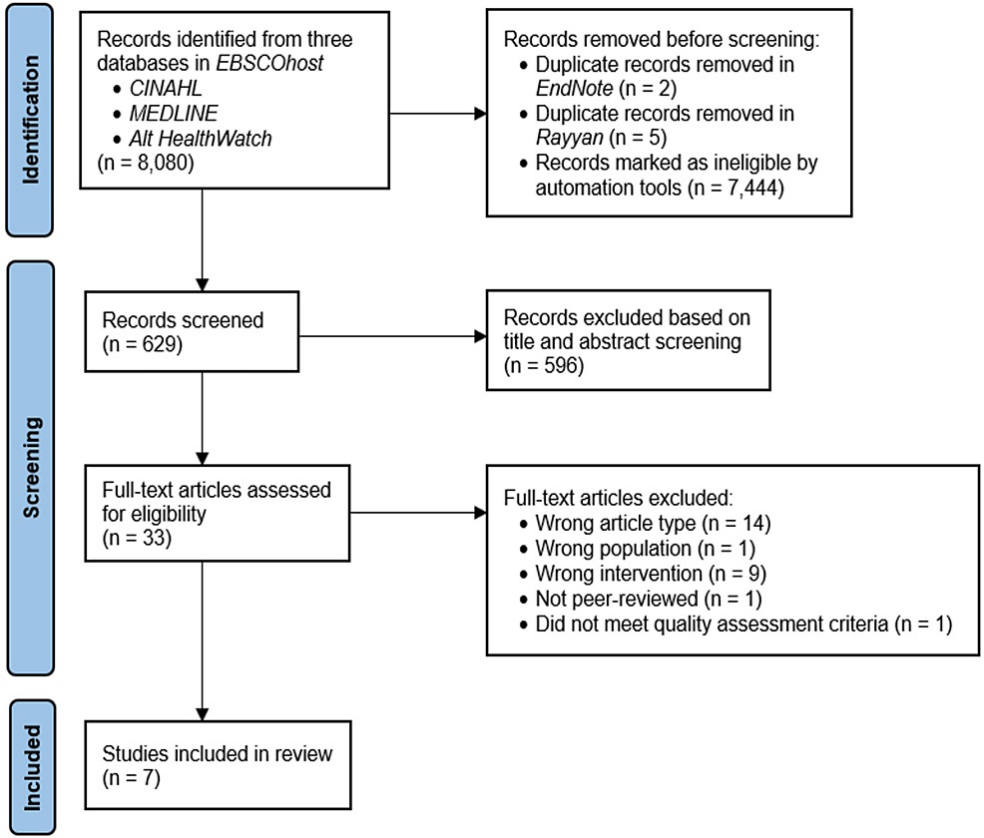
Preferred Reporting Items for Systematic Reviews and Meta-Analyses flow diagram of the study selection process.

The database search on EBSCOhost yielded 8,080 articles. After the removal of 7,444 articles identified as ineligible by EBSCOhost automation tools and seven duplicate records, the remaining 629 articles were exported to Rayyan for screening of title and abstract by all nine reviewers. An article was included if it had a minimum of five reviewers who voted to accept it. When two or more reviewers voted “maybe” on an article, a group discussion with all nine reviewers was held to make a final decision on whether to include it. A total of 596 articles were excluded based on title and abstract screening. The remaining 33 articles underwent full-text review and quality assessment independently by three reviewers per article using the corresponding Joanna Briggs Institute critical appraisal checklist. Articles that scored an average of 60% or greater on the quality assessment were included in the final analysis. Seven studies were ultimately included in this scoping review. Figure 1 illustrates the study selection process.

Results

All seven articles selected were published in peer-reviewed journals and included three cohort studies (retrospective, prospective, and observational), one prospective observational study, one prospective efficacy study, one prospective comparative diagnostic accuracy study, and one randomized control trial. The majority of the studies were conducted in the U.S. (n = 4) and the others in the United Kingdom (n = 1), Switzerland (n = 1), and Sweden (n = 1). A total of five fertility-tracking apps were assessed. Three articles reported data on the Natural Cycles app and the remainder on the following apps: Ava Fertility, Clearblue Connected, Ovia Fertility, and Dynamic Optimal Timing (DOT). The Appendix summarizes the data reported in each article.

User Data Collected to Provide Predictions

Natural Cycles aims to serve both family planning and contraception purposes. It requires users to purchase a thermometer to measure BBT. In addition to BBT, users are required to report menstruation data and have the option of presenting LH values if they purchase urine measurement kits [17-19]. Ava Fertility primarily aims to help users seeking conception. It uses a sensor bracelet (Ava bracelet) to continuously measure wrist skin temperature, heart rate, heart rate variability, and respiratory rate during sleep to determine ovulation. The Ava bracelet saves data every 10 seconds and synchronizes it to the app [20]. Clearblue Connected also mainly aims to help users seeking conception. To predict the fertile window, the app requires a urine hormone testing device to measure LH and estrone-3-glucuronide (E3G) levels, which connect to the app via Bluetooth, as well as self-reported menstrual data and intercourse frequency [21]. The only apps that do not require an external device are Ovia Fertility and DOT, which simply rely on self-reported user data [22,23]. Ovia Fertility aims to assist users with conception and provides day-specific probabilities of conception. Its algorithm is based on a statistical machine-learning model (i.e., Bayesian hierarchical model) in which self-reported data, such as cycle start and end dates, “spotting,” intercourse, and ovulation and pregnancy test results, are used to predict the start and end dates of menses. Users also have the ability to retroactively log days of menses [22]. In contrast, DOT mainly aims to help users with contraception and provides predictions of daily pregnancy risk and fertile window. Similar to Ovia Fertility’s algorithm, DOT’s algorithm also employs a Bayesian statistical model; however, in addition to the user’s self-reported data, DOT’s algorithm takes into account data from the WHO Ovulation Method Study (approximately 7,000 menstrual cycles), as well as clinical studies of variable fecundability in relation to ovulation. During the first few cycles using the app, DOT conservatively estimates the fertile window; as users enter more cycles, it better tailors the fertile window prediction [23]. Table 1 illustrates the marketed aim of and predictive factors collected by each app.

**Table 1:** Factors used by mobile applications to predict female fertility status.

Mobile application (iOs and Android)	Natural cycles	Ava Fertility	Clearblue Connected	Dynamic Optimal Timing (DOT)	Ovia Fertility
Aim of marketing	Contraception, conception	Conception	Conception	Contraception	Conception
Basal body temperature	Required		Optional		
Wrist skin temperature		Required			
Date of last menstrual period		Required	Required	Required	Optional
Usual cycle length			Required		Optional
Urine luteinizing hormone	Optional	Optional	Required		Optional
Urine estrone-3- glucoronide (E3G)			Required		
Intercourse frequency	Optional	Optional	Optional		Optional
Bleeding or spotting	Optional	Optional	Optional		Optional
Pregnancy test result	Optional	Optional			Optional
Other (optional)	Mood, sex drive, cervical mucus, pain, symptoms	Mood, cervical mucus			Mood, sleep, weight, cervical attributes, blood pressure, body symptoms, exercise, nutrition, medications

Reported Effectiveness

Three articles reported on Natural Cycles: two were published in 2021 and one was published in 2017 [17-19]. The 2017 study found a typical-use contraceptive failure rate of 8.3% (95% CI) over 13 cycles, a Pearl Index of 6.9 with typical use, and a Pearl Index of 1.0 with perfect use [17]. “Typical use” refers to the imperfect or inconsistent use of birth control in real-world environments, whereas “perfect use” refers to that observed in clinical studies with correct and consistent use of the method being studied. The 2021 study by Pearson et al. found a typical-use contraceptive failure rate of 7.2% (95% CI) over 13 cycles, a Pearl Index of 6.2 with typical use, and a Pearl Index of 2.0 with perfect use [18]. In addition, pregnancy probability was significantly higher in the 29 to 34-year-old age group than in the 34 to 45-year-old age group (8.8% vs. 5.0%; p = 0.0005) [19]. The other study published in 2021 assessed the app as an aid in family planning by measuring the time to pregnancy (TTP) and reported a median TTP of four cycles for the complete cohort with a cumulative pregnancy probability of 61% over six cycles and 74% over 12 cycles [18]. Of note, the cohort with the highest fecundability had an age below 35 years old, cycle length variation of less than five days, and reported sexual intercourse on more than 20% of days; the TTP of this relatively younger, eumenorrheic cohort was two cycles with a cumulative pregnancy probability of 88% over six cycles and 95% over 12 cycles [18].

Compared to Natural Cycles which uses BBT, Ava Fertility relies on wrist skin temperature. Zhu et al. compared wrist skin temperature measured by the Ava bracelet to daily oral BBT measurements taken with a device not associated with the app for detecting ovulation, and corroborated results using urine LH tests as the standard reference [20]. Wrist skin temperature proved to have a higher sensitivity than BBT in determining fertility (0.62 vs. 0.23; p < 0.001), albeit a lower specificity (0.26 vs. 0.70; p = 0.002) given a higher true-positive rate (54.9% vs. 20.2%) as well as higher false-positive rate (8.8% vs. 3.6%) [20].

While Natural Cycles and Ava Fertility rely on body temperature measurement, the Clearblue Connected algorithm uses LH and E3G urine levels to inform about the likelihood of conception [21]. In a randomized controlled trial, the test group was twice as likely to become pregnant in the first cycle of use than the control group which was not using any ovulation testing (25.4% vs. 14.7%; p < 0.001) [21]. The pregnancy rate of the test group was still greater than the control group after two cycles of use (36.2% vs. 28.6%; p = 0.026) with an odds ratio of 1.4 [21].

Regarding DOT and Ovia Fertility, the former has a typical-use contraceptive failure rate of 3.5% (95% CI) over six cycles [23], whereas the latter had no effectiveness data reported, although the authors did conclude that the self-reported user data in the app were consistent with established clinical knowledge of the menstrual cycle and fertility [22].

Discussion

The goal of this scoping review was to identify the various factors used by mobile apps marketed as a method of contraception and/or aid in family planning to predict female fertility status, as well as their documented effectiveness. The seven studies included in this review yielded information on five fertility-tracking apps: Natural Cycles, Ava Fertility, Clearblue Connected, Ovia Fertility, and DOT [17-23]. All apps provided predictions on fertility status throughout a woman’s menstrual cycle using proprietary algorithms and self-reported user data. Additionally, Natural Cycles, Ava Fertility, and ClearBlue Connected required an external device in conjunction with the app. Natural Cycles requires a thermometer to measure BBT, and urine LH testing is optional [17-19]. Ava Fertility uses the Ava bracelet to measure wrist skin temperature as the user sleeps [20]. Clearblue Connected requires urine LH and E3G testing [21]. Whereas Natural Cycles requires the user to enter BBT manually, the devices required by Ava Fertility and Clearblue Connected synchronize results with the app via Bluetooth automatically, which could minimize reporting errors.

Of the five apps, Natural Cycles has the most literature available [17-19]. It is also the first and currently the only app approved by the U.S. Food and Drug Administration (FDA) for contraception since August 2018 [24]. In 2017, Berglund et al. published Natural Cycles’ 13-cycle, typical-use contraceptive failure rate of 8.3% (95% CI), and in 2021, Pearson et al. reported a failure rate of 7.2% (95% CI) [17,19]. In addition, both studies reported a typical-use Pearl Index of 6.9 and 6.2, respectively. Berglund et al. included a considerably larger, more diverse study population: 22,785 women using the app with the intent of preventing pregnancy, including users from 37 different countries with a majority (79%) from Sweden and a mean (SD) age of 29.2 (5.0) [16]. On the other hand, Pearson et al. included a sample of 5,879 women with a comparable mean (SD) age of 30.1 (4.8) but only from the U.S., of whom 68.1% had a university degree level of education, and the motivation for using the app (contraception vs. conception) was not indicated [19]. The authors also noted that their effectiveness data were limited by user behavior, such as compliance with app recommendations based on predicted fertility status and consistency of data logging, which could not be feasibly verified given the setting of the study. They reported that only 40.9% of women followed through with 13 cycles of data collection and only 18% reported information on practices during the fertile window [19]. However, their more homogenous study population potentially avoided the effects of outliers.

Overall, the more commonly used predictive factors among the apps included a type of temperature measurement, such as BBT [17-19] or wrist skin temperature [20], and/or urine LH testing [17-19,21]. Urine LH levels are considered a standard reference in determining ovulation status [20]. Zhu et al. compared wrist skin temperature measured by the Ava bracelet to oral BBT measurements and corroborated results using urine LH tests. Wrist skin temperature proved to have a higher sensitivity than BBT in determining fertility (0.62 vs. 0.23; p < 0.001), but lower specificity (0.26 vs. 0.70; p = 0.002) [20]; therefore, it appears to be a more accurate approach for women seeking contraception rather than conception. The Ava bracelet received FDA approval in January 2021 as the first wearable fertility tracker [25]. Interestingly, Clearblue Connected incorporated urine E3G levels in addition to LH levels to determine fertile windows. As the only randomized controlled trial in this review, results from the first cycle of use by women wanting to conceive proved to be promising as the test group was twice as likely to become pregnant than the control group which was not using any ovulation testing (25.4% vs. 14.7%; p < 0.001) [21]. Compared to Ava Fertility, Clearblue Connected appears to be a more suitable option for women seeking to conceive rather than prevent pregnancy.

Ovia Fertility and DOT only rely on self-reported user data and both algorithms employ Bayesian statistical analysis to predict fertile window [22,23]. However, DOT’s algorithm, in addition to user-logged data, incorporates data from approximately 7,000 menstrual cycles from the WHO Ovulation Method Study, as well as clinical studies of variable fecundability in relation to ovulation [23]. Perhaps, this suggests greater applicability to a more general population of child-bearing age, including women with menstrual irregularities. On the other hand, Ovia Fertility’s algorithm only considers data logged by the user to make predictions; therefore, it seems to be a less viable option for someone who would find entering data daily challenging. Faust et al. did not report any effectiveness statistics on Ovia Fertility, although they concluded that the self-reported data from their study population were consistent with established clinical knowledge of the menstrual cycle and fertility [22]. Jennings et al. did, however, report that DOT has a six-cycle, typical-use contraceptive failure rate of 3.5% (95% CI) [23]; thus, DOT appears to be a reliable option for women interested in using a fertility-tracking app that does not require an external device.

Limitations of included studies

Most of the studies were funded by the company that owns the app and/or included at least one author receiving some form of financial compensation from the company, which suggests the potential for reporting bias. The Appendix details these potential conflicts of interest. While this industry funding may distort the authors’ objectivity in the report of app efficacy, the information gathered regarding the factors used by each app is less objective and, thus, likely unaffected by this. However, given the proprietary nature of the algorithms used to determine fertility, the studies did not give specific details regarding the method of calculation. Additionally, all studies were conducted in developed countries (U.S., n = 4; United Kingdom, n = 1; Switzerland, n = 1; Sweden, n = 1); therefore, the results may not apply to populations in developing countries. Given that the studies did not report details of the proprietary algorithms and the population data used to determine the algorithm, it is unclear if the algorithms can be generalized to world populations.

Most of the data collected by the apps are self-reported and therefore subject to error and inconsistency. All studies, except the randomized controlled trial conducted by Johnson et al., obtained their study population through convenience sampling, and thus most of the results in this review may not be generalizable. Some studies sampled women trying to get pregnant, while others sampled women trying to prevent pregnancy. Depending on the desired outcome, the users may have had different levels of motivation to comply with all apps’ requirements and thus had varying levels of reporting consistency. In addition, the general population may not be as motivated as either of these sampled populations; thus, it is difficult to determine if the efficacy is generalizable to the general population.

Limitations of the review process

The number of included studies in this review may have been limited for various reasons. For instance, only three databases were used, i.e., CINAHL, MEDLINE, and Alt HealthWatch. In addition, only articles published in English were included, given that all reviewers spoke and understood this language. It is also possible that the specific search terms used may have excluded articles that would have been relevant.

Future research and implications for clinical practice

Evidence-based knowledge on the effectiveness of fertility-tracking apps is limited, as well as research without the potential influence of commercial bias. To avoid possible conflicts of interest, more independent research is needed. Longitudinal studies and/or randomized controlled trials are needed to validate the efficacy of these fertility apps. In addition, head-to-head trials comparing apps to conventional contraceptive methods, such as oral contraception and or family planning and barrier methods, are necessary to determine the validity of these methods in communities that have these options. In addition, more ethnically, racially, and geographically diverse study populations are needed to increase generalizability. In particular, studies conducted in developing countries would be of great interest. Considering accessibility and cost-effectiveness, this method of contraception might improve fertility planning for families in low- to middle-income countries in which access to pharmaceutical contraception is limited. In addition, fertility apps can provide individuals with more autonomy over their health. This review provides valuable information for healthcare providers who are considering recommending fertility apps to their patients. In acknowledging the limitations of reported efficacy and by detailing the factors patients will be expected to provide, the review provides a framework with which healthcare providers can engage in shared decision-making with their patients. While the information in this review is not conclusive, future research in this area may serve to inform clinical guidelines and regulatory policy.

## Conclusions

Despite their rising popularity, evidence-based knowledge on the effectiveness of fertility-tracking apps as a method of contraception and family planning is limited. The user data collected by these apps varies between platforms and is largely self-reported; therefore, their predictive accuracy depends on the degree of user engagement. There were concerns about potential research bias as most studies included in this review were funded by the companies that own their respective apps. Additionally, the majority of the studies obtained their study population through convenience sampling, which limits the generalizability of the results. Despite these limitations, this review provides valuable information for healthcare providers to inform their recommendations to patients seeking non-hormonal, non-invasive contraception and family planning options.

## Appendices

**Table 2:** Data reported in included studies. U.S. = United States; BBT = basal body temperature; LH = luteinizing hormone; PI = Pearl Index; WST = wrist skin temperature; HR = heart rate; RR = respiratory rate; TTP = time to pregnancy; UK = United Kingdom; E3G = estrone-3-glucuronide; DOT = Dynamic Optimal Timing; WHO = World Health Organization

Study	Study design	Study aims	Study population	Mobile app and required device	Factors used by the app to predict fertility	Reported effectiveness	Aim of marketing	Potential conflicts of interest and study limitations
Pearson et al., June 2021 [19]	A prospective real-world cohort study	To assess the effectiveness of Natural Cycles at preventing pregnancy and describe the key demographics of current users	A convenience sample of 5,879 women in the U.S. aged 18-45 years; mean age 30.1±4.8; mean BMI 23.5±4.5; 86.1% reported being in a relationship; 68.1% had a university degree level of education	Natural Cycles (iOS and Android); Natural Cycles basal thermometer; optional urine LH testing	The algorithm relies on self-reported BBT, menstruation dates, and optional LH test results to provide a daily fertility status	13-cycle typical-use contraceptive failure rate of 7.2% (95% CI); one-year typical-use PI of 6.2, perfect-use PI of 2.0; age was associated with effectiveness: pregnancy probability was significantly higher between 29 and 34 years than 34 and 45 years (8.8% vs. 5.0%; p = 0.0005)	Contraception and conception	Conflicts of interest: Of the nine total authors, two are the scientists behind the app and founders of the company with stock ownership, four are employees of the company, and three serve on the medical advisory board of the company and have received an honorarium for participating in advisory boards and/or giving presentations for matters related to contraception and fertility regulation for Ferring, Exelgyn, and Mithra, which are all pharmaceutical companies; the study was funded by Natural Cycles USA Corp. Study limitations: Results were not generalizable given nonrandom sampling; 40.9% of women originally enrolled remained by the end of the study and only 33% had contributed 13 cycles of data; all data were self-reported; some women may have been using the app to conceive rather than prevent pregnancy
Zhu et al., June 2021 [20]	A prospective comparative diagnostic accuracy study	To compare accuracy in detecting ovulation between continuous monitoring of WST during sleep using the Ava Fertility Tracker bracelet 2.0 (Ava bracelet) and BBT measured orally using Lady-Comp; results from home-based urine LH tests using the ClearBlue Digital Ovulation Test were used as the standard reference	A convenience sample of 57 women in Switzerland aged 18-45 years; mean age 26.7 ± 4.2; mean BMI 22.5 ± 3.6; race 75% White	Ava Fertility (iOS and Android); Ava bracelet	The Ava bracelet uses sensor technology to continuously measure physiological data (WST, HR, HR variability, and RR) while the user sleeps	WST had higher sensitivity than BBT (0.62 vs. 0.23; p < 0.001) with a higher true-positive rate (54.9% vs. 20.2%) but also a higher false-positive rate (8.8% vs. 3.6%), which resulted in a lower specificity (0.26 vs. 0.70; p = 0.002)	Conception	Conflicts of interest: Of the nine total authors, one was an employee of Ava AG, one was a former employee, and one was a member of the advisory board. Study limitations: Relatively small sample size; results were not generalizable given non-random sampling; factors that could potentially influence temperature were not evaluated (e.g., sexual activity, exercise, food intake, alcohol consumption, sleep duration, and quality, and wake-up time)
Favaro et al., January 2021 [18]	An observational cohort study	To investigate the TTP, a biomarker of fecundability, of users of Natural Cycles	A convenience sample of 5,376 women trying to conceive aged 18–45 years with the majority living in Sweden (31%), the UK (29%), and the U.S. (14%); 46.3% between 30 and 34 years; 68.9% BMI of 18.5–24.9; 68.8% reported university degree as their maximum education level	Natural Cycles (iOS and Android); Natural Cycles basal thermometer; optional urine LH testing	The algorithm relies on self-reported BBT, menstruation dates, and optional LH test results to provide a daily fertility status	Six-cycle pregnancy probability of 61% (95% CI); 12-cycle pregnancy probability of 74% (95% CI); median TTP was four cycles; the highest fecundability was associated with an age of <35 years, cycle length variation of <5 days, and logging sexual intercourse on at least 20% of days	Contraception and conception	Conflicts of interest: Of the nine total authors, six were full-time employees of the company with shares, two were the founders of the company with shares, one previously received honorarium for presentations on the app, and media activities, and was the founder of the forum globalwomenconnected.com; the study was funded by Natural Cycles USA Corp; the study was also supported by the Intramural Research Program of the NIH, National Institute of Environmental Health Sciences. Study limitations: All data were self-reported; users had a higher educational level and lower BMI than would be expected in the general population given non-random sampling; close to 50% of users had characteristics associated with subfertility such as age >35 years, highly variable cycle length, or anovulatory cycles; relatively high dropout rates
Johnson et al., January 2020 [21]	An open, home-based, randomized control trial	To evaluate the effectiveness of the Clearblue Connected Ovulation Test System as a method of fertility tracking to inform users of ideal timing for intercourse and thus increase the likelihood of conception	844 women from England, Wales, or Scotland aged 18–40 years trying to conceive; the median age of 30 in both groups; test group BMI 26.43 ± 5.65; control group BMI 26.48 ± 5.68	Clearblue Connected (iOS and Android); Clearblue Connected Ovulation Test System, a home-based urine hormone testing device	Predicts fertile window based on urine LH and E3G levels, self-reported menstrual cycle length, and intercourse frequency	The test group was twice as likely to become pregnant in the first cycle of use than the control group which was not using any ovulation testing (25.4% vs. 14.7%; p < 0.001), odds ratio of 2.0 (95% CI); pregnancy rate of the test group was still greater than the control group after two cycles of use (36.2% vs. 28.6%; p = 0.026), odds ratio of 1.4 (95% CI)	Conception	Conflicts of interest: The study was funded by Swiss Precision Diagnostics (SPD) Development Company, owner of the Clearblue brand; of the six total authors, four were employees of the SPD Development Company, one was a paid scientific consultant, and one was an advisory board member. Study limitations: Lack of evidence of longer-term use given that the study only evaluated the test system across two menstrual cycles; part of the data collected were self-reported
Faust et al., July 2019 [22]	A retrospective cohort study	To assess the validity of self-reported data from Ovia Fertility by comparing it to established clinical knowledge of the menstrual cycle and fertility	A convenience sample of 98,903 women (225,596 menstrual cycles) who are residents of the U.S. aged 18–45 years and reported “just started trying to conceive”; 66% no previous children; 70% between 25 and 34 years; race 78% White	Ovia Fertility (iOS and Android); optional ovulation testing	The algorithm is based on a statistical machine-learning model (Bayesian hierarchical model) in which self-reported data, such as cycle start and end dates, “spotting,” intercourse, and ovulation/pregnancy test results, are used to predict the start and end dates of menses and fertile window	Effectiveness was not reported, but authors concluded that self-reported data in the app were consistent with established clinical knowledge of the menstrual cycle and fertility	Conception	Conflicts of interest: Of the seven total authors, one received consulting fees from the app company for statistical analysis consulting. Study limitations: Results were not generalizable given non-random sampling; all data were self-reported
Jennings et al., January 2019 [23]	A prospective efficacy study	To assess the six-cycle perfect- and typical-use efficacy of the DOT app	A convenience sample of 629 women in the continental U.S. already using the app on an Android phone to prevent pregnancy aged 18–35 years; 36.5% between 25 and 29 years; 48% reported being in a relationship for >3 months; race 54.7% White	DOT (iOS and Android)	The algorithm is based on Bayesian statistical analysis of about 7,000 menstrual cycles from the WHO Ovulation Method Study, as well as clinical studies of variable fecundability in relation to ovulation, and calculates fertile window using self-reported period start dates	Of 629 women, 15 became pregnant resulting in a six-cycle typical-use contraceptive failure rate of 3.5% (95% CI); the perfect-use contraceptive failure rate was not calculated given that all pregnancies occurred due to incorrect use; the app is calibrated to provide a contraceptive failure rate of no more than 1–3 per 100 woman years of perfect use	Contraception	Conflicts of interest: Of the five total authors, four were employed by the Institute for Reproductive Health (IRH) at Georgetown University (GU), which is recipient of a grant from the U.S. Agency for International Development that supports this study; a patent application for the app has been filed by Cycle Technologies, a company that is owned by a family member of the director of the IRH, but none of the authors or any other employee of GU received any financial benefit from Cycle Technologies. Study limitations: Results were not generalizable given non-random sampling; all data was self-reported
Berglund et al., January 2017 [17]	A prospective observational study	To assess the effectiveness of Natural Cycles as a form of contraception with perfect-use versus typical-use PI	A convenience sample of 22,785 women using the app with the intent of preventing pregnancy between August 1, 2014, and August 1, 2016; users from 37 different countries with a majority (79%) from Sweden; mean age 29.2 ± 5.0	Natural Cycles (iOS and Android); Natural Cycles basal thermometer; optional urine LH testing	The algorithm relies on self-reported BBT, menstruation dates, and optional LH test results to provide a daily fertility status	13-cycle typical-use contraceptive failure rate of 8.3% (95% CI); typical-use PI of 6.9 (95% CI); perfect-use PI of 1.0 (95% CI)	Contraception and conception	Conflicts of interest: Of the six total authors, two were the scientists behind the app and founders of the company with stock ownership, one was an employee, two served on the medical advisory board and had received an honorarium for participating in advisory boards and/or giving presentations for matters related to contraception and fertility regulation for MSD/Merck, Bayer AG, Gedeon Richter, Exeltis, Actavis, Ferring, Exelgyn, and Mithra which are all pharmaceutical companies; the study was funded by Natural Cycles Nordic AB and partial support was provided from an infrastructure grant for population research from the Eunice Kennedy Shriver National Institute of Child Health and Human Development of the National Institutes of Health. Study limitations: Results were not generalizable given nonrandom sampling; all data were self-reported
